# MDR Acinetobacter baumanni in the short term and long term acute care setting

**DOI:** 10.1186/1753-6561-5-S6-O26

**Published:** 2011-06-29

**Authors:** JL Whitaker

**Affiliations:** 1 Infection Prevention and Control, University Community Hospital, Tampa, FL, USA

## Introduction / objectives

Multi-drug resistant Acinetobacter baumannii is a health care-associated pathogen that can live for months in both a wet and dry environment. The high prevalence of this organism in the hospital environment results in colonization of the skin and respiratory tract in the patient population, which can lead to development of infection. Determine if development of an admission screening protocol and 10% hypochlorite disinfection will significantly reduce the incidence of health care-associated infections in the patient population.

## Methods

A case-only study was conducted over a 12-month period. Interventions used to reduce the incidence of healthcare associated Acinetobacter baumannii included 10% hypochlorite disinfection, hand hygiene, special contact isolation for suspected and confirmed cases, educational tool for clinicians, patient and visitors, daily isolation rounds, automated report functions, and standardized nursing unit isolation practices. Pulse-field gel electrophoresis was performed on all isolates to determine if there was a common genotype among the patient population.

## Results

There were a total of eighty-five (85) isolates collected during the 12-month period. 52 (61%) were healthcare-associated and 33 (39%) were community acquired. In the first month of implementation of a new protocol to collect respiratory specimens on admission from other acute care facilities, there was an 87.5 % reduction in healthcare-associated isolates.

## Conclusion

A combination of an admission screening protocol of patients transferred from other acute care facilities, implementation of a 10% hypochlorite disinfection protocol and isolation of those patients at time of admission until negative culture results can prevent transmission of healthcare-associated and community acquired MDR Acinetobacter in a healthcare entity.

## Disclosure of interest

None declared.

